# Lung microbiome alterations in patients with anti-Jo1 antisynthetase syndrome and interstitial lung disease

**DOI:** 10.3389/fcimb.2023.1321315

**Published:** 2023-12-05

**Authors:** Teresa Quintero-Puerta, Juan Alberto Lira-Lucio, Ramcés Falfán-Valencia, Ángel E. Vega-Sánchez, Eduardo Márquez-García, Mayra Mejía, Brandon Bautista-Becerril, Jorge Rojas-Serrano, Espiridión Ramos-Martínez, Ivette Buendía-Roldán, Gloria Pérez-Rubio

**Affiliations:** ^1^HLA Laboratory, Instituto Nacional de Enfermedades Respiratorias Ismael Cosío Villegas, Mexico City, Mexico; ^2^Interstitial Lung Disease and Rheumatology Unit, Instituto Nacional de Enfermedades Respiratorias Ismael Cosío Villegas, Mexico City, Mexico; ^3^Subdirección de Investigación Biomédica, Instituto Nacional de Enfermedades Respiratorias Ismael Cosío Villegas, Mexico City, Mexico; ^4^Experimental Medicine Research Unit, Facultad de Medicina, Universidad Nacional Autónoma de México, México City, Mexico; ^5^Laboratory of Translational Research in Aging and Pulmonary, Instituto Nacional de Enfermedades Respiratorias Ismael Cosío Villegas, Mexico City, Mexico

**Keywords:** lung microbiome, 16S ribosomal subunit, antisynthetase syndrome, interstitial lung disease, *Veillonella*

## Abstract

**Aim:**

To characterize the lung microbiome in the bronchoalveolar lavage fluid (BALF) of patients with Antisynthetase Syndrome (ASSD) according to anti-Jo1 autoantibody positivity and evaluate the correlation with differential cell count and other bacterial genera in BALF.

**Methods:**

We sequenced the 16S ribosomal RNA gene in the BALF of anti-Jo1-positive (JoP, n=6) and non-Jo1-positive (NJo, n=17) patients, and the differential cell count in BALF was evaluated. The Spearman’s correlation was calculated for the quantitative variables and abundance of bacterial species.

**Results:**

The *Veillonella* genus showed a significant decrease (p<0.01) in JoP (2.2%) in comparison to NJo (4.1%) patients. The correlation analysis showed several high (rho ≥ ± 0.7) and significant (p < 0.05) correlations. We analyzed the results obtained for the *Veillonella* genera and other study variables. The JoP group showed that the abundance of *Veillonella* had a high negative correlation with macrophages (rho = - 0.77) and a positive correlation with eosinophils (rho = 0.77), lymphocytes (rho = 0.77), and *Prevotella* (rho = 1).

**Conclusions:**

The lung microbiome in ASSD patients differs and may affect cell composition, contributing to lung damage mechanisms. The presence of anti-Jo1 autoantibodies showed a low abundance of *Veillonella*. This genus had a strong and positive correlation with *Prevotella* abundance and levels of eosinophils and lymphocytes, and it showed a strong negative correlation with the percentage of macrophages.

## 1 Introduction

Antisynthetase syndrome (ASSD) is a rare autoimmune disease (1.5 cases per 100, 000) that commonly exists with extra-muscular manifestations, including fever, Raynaud’s syndrome, arthritis, mechanic’s hands, and interstitial lung disease (ILD) (Hamaguchi et al., 2013; Marie et al., 2013b; Rojas-Serrano et al., 2015; Pinal-Fernandez et al., 2017). This pathology is characterized by the presence of autoantibodies against various aminoacyl-transfer RNA (tRNA) synthetases (Witt et al., 2016; Pinal-Fernandez et al., 2017), including anti-Jo1 (most common), anti-PL7, anti-PL12, anti-OJ, anti-EJ, anti-KS, anti-Zo, anti-SC, anti-JS, and anti-YRS (Lega et al., 2010; Aggarwal et al., 2014; Rojas-Serrano et al., 2015; Pinal-Fernandez et al., 2017; Ponce-Gallegos et al., 2020). Independent of the autoantibody subtype, the leading predictive factors for a progressive pulmonary disease are a low diffusing capacity of the lungs for carbon monoxide (DL_CO_) at diagnosis, a low forced vital capacity (FVC) at baseline evaluation (Rojas-Serrano et al., 2015), and muscle weakness (Kamiya et al., 2018; Gasparotto et al., 2019; Liu et al., 2020). The abnormalities most often observed using high-resolution computed tomography (HRCT) are organized pneumonia (OP), followed by nonspecific interstitial pneumonia (NSIP) (González-Pérez et al., 2020).

Differences exist between anti-Jo1-positive (JoP) and non-Jo1-positive (NJo) patients, with the latter often demonstrating ILD in the absence of muscle involvement and having worse survival (Aggarwal et al., 2014); however, JoP patients present higher Goh inflammation score (Lega et al., 2010; Aggarwal et al., 2014; Rojas-Serrano et al., 2015; Pinal-Fernandez et al., 2017; Ponce-Gallegos et al., 2020). The Goh score is a semi-quantitative CT (SQCT) assessment that can estimate inflammation associated with ILD (Rojas-Serrano et al., 2015). Lung inflammation is a mechanism that has a crucial role in tissue repair, but if unregulated, it can be an underlying cause of chronic lung diseases (Huffnagle et al., 2017). Interactions between the host and lung microbiome, primarily at the mucosal surfaces, are fundamental to developing and regulating immunity mechanisms (Neish, 2014).

The lungs are constantly exposed to diverse communities of microbes from the oropharynx and other sources. The most abundant phyla of healthy lungs are Bacteroidetes and Firmicutes. At the genera level, *Prevotella, Veillonella*, and *Streptococcus* are predominant (Dickson et al., 2016). The disruption of the lung microbiome (dysbiosis) contributes to acute inflammation, progression, or exacerbations in diseases like asthma, chronic obstructive pulmonary disease (COPD), and interstitial lung disease (ILD) (Ren et al., 2018; Paudel et al., 2020). The most frequent ILD is idiopathic pulmonary fibrosis (IPF), where there is clear evidence of the microbiome’s contribution to collagen deposition in the lungs (O’Dwyer et al., 2019). In the microbiome of patients with stable IPF predominates *Streptococcus, Prevotella, Veillonella, Haemophilus*, and *Pseudomonas*. In contrast, IPF patients with acute exacerbation demonstrate increased *Campylobacter* sp. and *Stenotrophomonas* sp., associated with a significant decrease in *Veillonella* sp (Molyneaux et al., 2017). Disease progression has been associated with a high abundance of *Streptococcus* and *Staphylococcus* and a loss of diversity in the lungs (Han et al., 2014).

The dysbiosis of the lung microbiome is a major factor contributing to developing and progressing ILD associated with dermatomyositis, IPF, and rheumatoid arthritis. However, in ASSD, the lung microbiome has not been explored. This study aimed to characterize the lung microbiome in the bronchoalveolar lavage fluid (BALF) of patients with ASSD according to the presence or absence of anti-Jo1 autoantibodies and to evaluate correlation with differential cell count in BALF.

## 2 Material and methods

### 2.1 Patient details

We included 23 patients with a confirmed diagnosis of ASSD who were recruited from the Interstitial Lung Disease and Rheumatology Unit of Instituto Nacional de Enfermedades Respiratorias Ismael Cosío Villegas (INER) in Mexico. A multidisciplinary group (pulmonologists, rheumatologists, and radiologists) performed the diagnosis. All patients were newly diagnosed. We registered the patients’ demographic variables, tobacco consumption, results of pulmonary function tests (including spirometry and diffusion capacity of the lungs for carbon monoxide, DL_CO_), levels of creatine-phosphokinase (CPK), differential cell count in BALF, clinical manifestations, and autoantibody patterns, which include anti-Mi-2α, anti-Mi-2β, anti-TIF1γ, anti-MDA5, anti-NXP2, anti-SAE1, anti-Ku, anti-PM-Scl100, anti-PM-Scl75, anti-Jo1, anti-SRP, anti-PL7, anti-PL12, anti-EJ, anti-OJ, and anti-Ro-52 (EUROIMMUN. Lübeck, Germany), as well as specific HRCT (Rojas-Serrano et al., 2015; González-Pérez et al., 2020). All participants were invited to donate their remnant BALF sample, signed an informed consent letter, and were provided with a personal data protection document. The exclusion criteria were the presence of an infectious disease, the use of antibiotics or immunosuppressive medications in the past three months, and evidence of acute upper respiratory symptoms in the previous four weeks. The patients were divided into anti-Jo1-positive (JoP) and non-Jo1-positive (NJo) groups. The Institutional Committees for Research, Biosecurity, and Ethics in Research of the INER approved this study (approval number B28-18).

### 2.2 BALF processing and bacterial DNA extraction

The BALF samples were centrifuged at 3,000 rpm at 20˚C for 10 minutes, and the supernatant was stored at -30°C. The cell pellet was employed for bacterial DNA extraction and purified using the ZymoBIOMICS™ DNA Miniprep Kit (Zymo Research Corp. CA, EU), following the manufacturer’s instructions for liquid samples (200 µL). DNA was quantitated using the Qubit dsDNA High Sensitivity (HS) Assay Kit (Invitrogen) and visually assessed for integrity via electrophoresis on a 1% agarose gel.

### 2.3 16S Metagenomic sequencing library preparation

We prepared the libraries for targeted amplicon sequencing following the “16S Metagenomic Sequencing Library Preparation” guide (Part# 15044223 Rev. B, Illumina). We used the primer pair sequence (forward primer = 5’ TCGTCGGCAGCGTCAGATGTGTATAAGAGACAGCCTACGGGNGGCWGCAG; reverse primer = 5’ GTCTCGTGGGCTCGGAGATGTGTATAAGAGACAGGACTACHVGGGTATCTAATCC) for the V3 and V4 regions of the 16S ribosomal RNA gene (16S rRNA) and Nextera XT indices. The sequencing was run on the MiSeq platform using paired 300 bp reads and MiSeq v3 reagents. We employed MiSeq Control Software v 3.1.1.13 (Illumina) to generate the FASTQ file. We included two control saline samples to identify sources of bacterial contamination; these controls were treated under the same conditions as the BALF.

### 2.4 Bioinformatics analysis of sequencing results

We analyzed the FASTQ file using RStudio (R Studio, 2019), with the packages DADA2 (Callahan et al., 2016a), Phyloseq (McMurdie and Holmes, 2013), DESeq2 (Love et al., 2014), ggplot 2 (Lin Pedersen, 2019), and microbiome (Lahti et al., 2017), and followed the workflow of Bioconductor (Callahan et al., 2016b) and CastroLab (Castro Nallar, 2020). To assign taxonomy, we used the database of silva nr v138 and 97% similarity level (Quast et al., 2013). Alpha diversity was evaluated using the Chao1, Shannon, and Simpson indexes; beta diversity was evaluated using Bray–Curtis dissimilarity and principal coordinate analysis (PCoA). The readings were normalized to obtain the amplicon sequence variants (ASVs).

### 2.5 Evaluation of species of *Veillonella*

According to the results obtained using 16S metagenomics, we selected three bacterial species belonging to *Veillonella* with clinical relevance. The specificity of each primer set and probe was evaluated using the basic local alignment search tool (BLAST) (https://blast.ncbi.nlm.nih.gov/Blast.cgi) ([[NoAuthor]]) (**Supplementary Table 1**). A real-time PCR assay was performed using the StepOnePlus system (Applied Biosystems™). We used TaqMan custom assay to evaluate *V. parvula* and *V. dispar*. The reactions were performed using a total volume of 10 µl that contained 5 µl of Maxima Probe/ROX qPCR Master Mix (2X) (Thermo Scientific, Vilnius, Lithuania), 0.5 µl each of the forward and reverse primers (final concentration, 300 µM), 0.03 µl of TaqMan custom probe (final concentration 70 µM), 2 µl of the template DNA solution (20 ng/µl), and 2 µl of nuclease-free water. The thermal cycling conditions for all real-time PCR assays were as follows: 1 cycle at 50°C for 10 minutes, 1 cycle at 95°C for 10 minutes, followed by 45 cycles at 95°C for 15 seconds and at 60°C for 1 minute. For *V. atypica*, we used SYBR green qPCR in a total volume of 25 μL that contained 4 μl of the template DNA solution (20 ng/μL), 3.5 μL of nuclease-free water, 12.5 mL of Maxima SYBR Green/ROX qPCR Master Mix (2X) (Thermo Scientific, Vilnius, Lithuania), and 3 μL of the forward and 3 μL of reverse primers (final concentration 375 μM). The thermal cycling conditions for all real-time PCR assays were 1 cycle at 95°C for 10 minutes, 40 at 95°C for 30 seconds, and 1 at 56°C for 1 minute.

### 2.6 Statistical analysis

We employed the Shapiro–Wilk test to assess the normality of demographic characteristics, smoking status, pulmonary function test results, cells present in BALF, clinical manifestations, autoantibody profile, and HRCT pattern. For quantitative variables, we presented the means and standard deviations or medians and interquartile ranges (IQR); according to the distribution of the variables, we used parametric or non-parametric tests. The categorical data were analyzed using a contingency table and Fisher’s exact test. The identified ASVs were compared in both study groups; we employed a negative binomial generalized linear model (GLM) and obtained maximum likelihood estimates for an ASV log-fold change between two study groups; the p-value was adjusted based on the false discovery rate (FDR) measured using the Benjamin–Hochberg method. Alpha diversity was assessed using the Mann–Whitney U test. Beta diversity was assessed using the Wilcoxon signed-rank test. A p-value < 0.05 or an FDR < 0.05 was considered statistically significant. Spearman’s correlation was determined for the quantitative variables and abundance of bacteria. The results were presented using the ggplot2 package (Wickham, 2016) and corrplot (Wei and Simko, 2021). The dataset supporting the results of this article has been deposited in the NCBI Short Read Archive database under BioProject accession code PRJNA715451.

## 3 Results

### 3.1 Patients

We included 23 ASSD patients; six were JoP and 17 NJo (**Table 1**). Age and sex did not differ significantly between the two groups; however, JoP patients were older in comparison to the NJo group (62 vs. 55 years), and the female sex was predominant in both groups (83.3% vs. 58.3%) compared to the male sex (16.7% and 41.7%). The proportion of smokers was similar (33.3% vs. 35.3%), and the tobacco index was higher in JoP patients compared to NJo patients (7.1 pack-years vs. 4.5 pack-years), although this difference was not statistically significant. DLCO was lower in JoP patients compared to NJo patients, but there was no statistical difference (42% vs. 49%). Both groups had moderate DL_CO_ reduction (Ponce et al., 2022). Patients with JoP status had a smaller value in the 6MWT than NJo patients (120 *vs*. 405, respectively, p=0.01). Patients JoP showed high levels of CPK compared to the NJo group (242.5 vs. 67.5 U/L, respectively, p=0.001). The tomography patterns were inflammatory in both groups (JoP = 66.7% and NJo = 70.6%). The inflammatory component was defined based on ground-glass opacities, while the fibrotic component was defined based on reticular opacities (Rojas-Serrano et al., 2015). In the BALF samples, macrophages and lymphocytes were the predominant cells.

**Table 1:** Demographic and clinical characteristics of the patients in this study.

Variable	JoP (n = 6)	NJo (n = 17)	p-value
Age (years)	62 (57-75)	55 (49-67)	0.23
Female sex, n (%)	(5) 83.3	(10) 58.3	0.36
Male sex, n (%)	(1) 16.7	(7) 41.7	
BMI (kg/m^2^)	26 (24 – 30)	26 (25 – 28)	0.86
Smoking (%)	33.3	35.3	1.00
TI (pack-years)	7.1 (5.4 – 8.8)	4.5 (1.1 – 8.1)	0.64
FVC (%)	52 (43 – 56)	63 (53 – 74)	0.27
FEV_1_ (%)	57 (53 – 70)	61 (53 – 78)	0.67
FEV_1_/FVC	86.6 (85.4 – 87.2)	83.0 (76.8 – 86.6)	0.07
DL_CO_ % (not adjusted)	42.0 (35.0 – 60.0)	49.0 (43.5 – 64.0)	0.34
6MWT (meters)	120.0 (120.0 – 140.0)	405.5 (356.2 – 485.5)	**0.010**
CPK (U/L)	242.5	67.5	**0.001**
Tomography patterns (F/I) (%)	33.33/66.7	29.4/70.6	1.00
Differential cell count in BAL (%)			
Macrophages	72.2 (48.2 - 90.7)	77.2 (68.7 - 84.8)	0.89
Lymphocytes	42.0 (23.7 - 54.0)	20.8 (12.1 - 28.5)	0.63
Eosinophils	1.5 (1.0 - 3.0)	0.3 (0.0 - 1.1)	0.21
Neutrophils	1.0 (0.5 - 1.5)	0.2 (0.0 - 1.0)	0.60

JoP, patients with anti-Jo1-positive autoantibodies; NJo, patients with no anti-Jo1-positive autoantibodies; BMI, body mass index; TI, tobacco index; FVC, forced vital capacity; FEV_1_, forced expiratory volume in the first second; DL_CO_, diffusing capacity of the lungs for carbon monoxide; 6MWT, six-minute walking test; CPK, creatine-phosphokinase kinase; F, fibrotic; I, inflammatory; NA, not available. The values are presented as medians with interquartile ranges or percentages. The p-values obtained using Mann–Whitney U and Fisher’s exact tests were used to compare the groups.

The most frequent clinical manifestations in all patients were mechanic’s hands (43.5%), dyspnea (43.5%), and cough (43.5%). In addition, only the NJo group presented fever (35.3%) and weight loss (23.5%) (**Supplementary Table 2**). The patients in this study showed an overlap of antibodies typical of myositis and sclerosis. JoP patients showed a predominance of anti-OJ (33.3%), anti-MI-2β (33.3%), and anti- Th/To (33.3%), whereas NJo patients showed a predominance of anti-Ro52, anti-PL7, anti-PL12 (35.3% in each autoantibody), and Th/To (23.5%) (**Supplementary Table 3**).

### 3.2 Composition of the lung microbiome in ASSD patients

**Figure 1:**
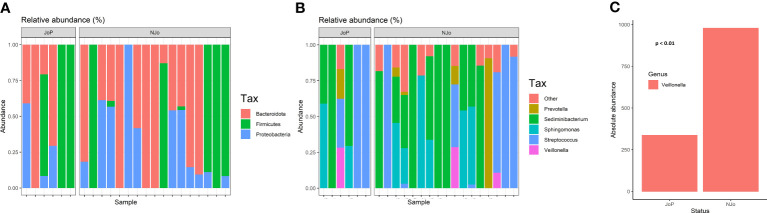
Composition of the lung microbiome in antisynthetase syndrome (ASSD) patients: **(A)** relative abundance of different phyla in both study groups; **(B)** top five genera in ASSD patients (according to relative abundance); and **(C)** Absolute abundance of *Veillonella* in JoP and NJo patients. This genus is lower in JoP patients (2.2%) than in NJo patients (4.1%).

We obtained 115 ASVs, and after performing quality control, we identified 10 ASVs with an abundance of >1,000 reads present in ≥ 10% of the samples analyzed. The datasets analyzed in this study (FASTQ file) can be found in online repositories (Sequence Read Archive, SRA; submission number: SUB9326500). The analysis of alpha diversity showed that there were no significant differences in the Shannon (p = 0.92), Simpson (p = 0.43), and Chao1 (p = 1) indexes (**Supplementary Figure 1**) between JoP and NJo groups. Beta diversity showed a statistically significant difference in divergence (p = 0.016), and using PCoA, we observed that overlap existed between the JoP and NJo groups (**Supplementary Figures 2A, B**). Both groups of patients predominantly showed phyla Firmicutes (71.32%), Bacteroidota (17.11%), and Proteobacteria (11.29%) (**Figure 1A**). The top five genera in all patients were *Streptococcus* (57.8%), *Sediminibacterium* (11.34%), *Veillonella* (8.92%), *Sphingomonas* (6.02%), and *Prevotella* (5.77%) (**Figure 1B**). A comparison of relative abundance in the two groups identified a statistically significant difference in the relative abundance of *Veillonella* (p < 0.01); this genus showed a low abundance in JoP patients (2.2%) in comparison to NJo (4.1%) patients (**Figure 1C**).

**Figure 2:**
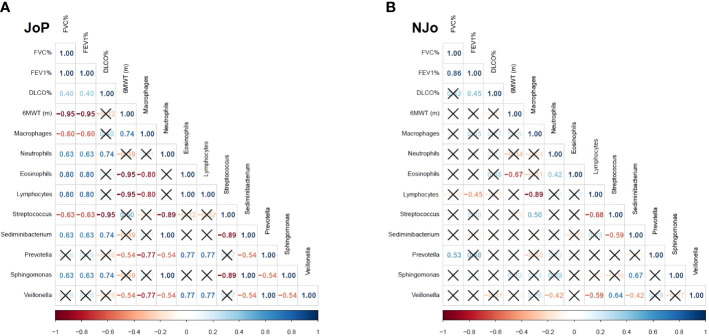
Spearman’s correlation in antisynthetase syndrome (ASSD) patients: **(A)** correlation analysis in the JoP group and **(B)** correlation analysis in the NJo group. Only the rho coefficients of significative correlations are shown.

The correlation analysis showed several high (rho ≥ 0.7) and significant (p < 0.05) correlations. We analyzed the results obtained for the *Veillonella* genus and other study variables. In the JoP group, *Veillonella* had a high negative correlation with macrophages (rho = - 0.77) and a positive correlation with eosinophils (rho = 0.77), lymphocytes (rho = 0.77) and *Prevotella* (rho = 1). In contrast, NJo patients did not show high correlations (**Figures 2A, B**).

Identifying specific species of *Veillonella* showed that *V. parvula*, *V. dispar*, and *V. atypica* had no significant difference between the study groups. We observed more patients who were positive for *V. dispar* in the NJo group (40.7%) compared to the JoP group (25.0%) (**Table 2**).

**Table 2:** Species identification in the study sample.

*Veillonella*	JoP (n=6)	NJo (n=17)	p-value
***parvula* (%)**	37.5	37.0	0.64
***dispar* (%)**	25.0	40.7	0.34
***atypica* (%)**	87.5	96.3	0.94

JoP, anti-Jo1-positive patients; NJo, non-Jo1-positive patients (NJo); p-value based on Fisher’s exact test.

## 4 Discussion

Previous reports have evaluated the lung microbiome and its influences on the course of various diseases, like asthma, cystic fibrosis, and chronic obstructive pulmonary disease; however, this is the first study to characterize the bacterial communities in patients with ASSD and ILD, which is the most frequent and severe extra-muscular manifestation (Gasparotto et al., 2019). The present study used the presence or absence of anti-aminoacyl-tRNA synthetase (anti-Jo1) autoantibodies with the strongest association with the development and severity of ILD in ASSD (Cavagna et al., 2019) to stratify the study patients.

We included a population of patients with ASSD. There were no significant differences in age, sex, BMI, TI, or pulmonary function test (FVC, FEV1, FEV1/FVC, and DL_CO_). Patients in the anti-Jo1-positive group walked significantly shorter distances during the 6MWT compared to the anti-Jo1-negative patients (p=0.01, 120 m vs. 405.5 m, respectively). The anti-Jo1-positive group had a tendency to show decreased lung function compared to the anti-Jo1-negative group; this finding was similar to that reported in a Mayo Clinic cohort study, where 53% of its patients with anti-Jo1-positive ASSD had decreased lung function, regardless of treatment (Zamora et al., 2016). In Caucasian patients, the functional outcomes of the anti-Jo1 patients with ASSD were poor, as shown by the marked reduction of activities evaluated according to the disability scale of the Health Assessment Questionnaire (HAQ > 0.75) (Marie et al., 2013a). In our study group, ASSD patients positive for anti-Jo1 antibody present more important muscle involvement, represented by higher levels of CPK (Ponce-Gallegos et al., 2020).

The predominant tomographic pattern was inflammatory, regardless of autoantibodies classification (66.7% in anti-Jo1-positive and 70.6% in anti-Jo1-negative patients). Cells showing a higher abundance in BAL were macrophages and lymphocytes. These findings correspond to the findings previously reported in ASSD (Lang et al., 2021) and other interstitial diseases, such as rheumatoid arthritis associated with ILD (Lee et al., 2005).

Interestingly, no significant difference was found in alpha diversity (Chao1, Shannon, and Simpson indices); we assume this is because both groups were patients diagnosed with ASSD. The loss of bacterial diversity has been linked to pulmonary diseases with increased profibrotic and proinflammatory cytokines (O’Dwyer et al., 2019), contributing to the severe outcomes of diseases such as COPD, asthma, cystic fibrosis, and idiopathic pulmonary fibrosis (Tunney et al., 2013; Yagi et al., 2021). The alpha diversity of the lung microbiome did not show differences between dermatomyositis and rheumatoid arthritis associated with ILD (Lou et al., 2022) or compared with patients with rheumatoid arthritis and sarcoidosis (Scher et al., 2016).

In the analysis of beta diversity, we found a divergence between the two study groups (p=0.016); this is probably due to the heterogeneity in the clinical manifestations of ASSD, as well as the overlap in autoantibodies that exists in the participants, regardless of the presence or absence of anti-Jo1 positivity.

At the phylum level, the lung microbiome of patients with ASSD comprises Firmicutes, Bacteroidota, and Proteobacteria. In healthy individuals, previous reports indicate a predominance of the genera *Prevotella*, *Streptococcus*, *Veillonella*, *Neisseria*, *Haemophilus*, and *Fusobacterium* (Segal et al., 2016; Yu et al., 2016). In our study, patients with ASSD showed a predominance of *Streptococcus*, *Sediminibacterium*, *Veillonella*, *Sphingomonas*, and *Prevotella*; the genus *Veillonella* was found in lower abundance in the JoP group compared to the NJo group (p<0.01). This bacterial genus is anaerobic and can tolerate environments with higher oxidative stress because all species of *Veillonella* have the *rbr* gene that codes for ruberythrins; these enzymes have an antioxidant mechanism that gives them the ability to catalyze hydrogen peroxide and, consequently, reactive oxygen species in the microenvironment decrease (Zhou et al., 2017).

Patients in the JoP group showed a very strong positive correlation between the abundance levels of *Veillonella* and *Prevotella*, which are anaerobic bacteria. In cystic fibrosis, it has been reported that *Prevotella* can produce short-chain fatty acids (acetic, i-butyric, 2-methyl butyric, and i-valeric) that activate epithelial cells in the airways to produce cytokines (Mirković et al., 2015), act as chemotactic agents for neutrophils (Vinolo et al., 2011), and affect phagocytosis (Vinolo et al., 2009). There is also a strong positive correlation between the levels of *Sediminibacterium* and *Sphingomonas*. Previous *in vitro* assays have shown that *Sphingomonas* can induce apoptosis in human lung cells via the activation of caspases 3 and 9 (Asghar et al., 2021). *Sediminibacterium* is abundant in the blood of patients with type 2 diabetes (Qiu et al., 2019); however, it has not been reported in lung diseases. In addition, a strong negative correlation was found between the levels of *Streptococcus* and neutrophils; previous research with mouse models has shown that the M1 protein of *Streptococcus* forms complexes with fibrinogen that binds to β2 integrin on the surface of neutrophils, which causes degranulation, increased vascular permeability, lung damage, and remodeling (Soehnlein et al., 2008).

As for ASSD patients who were anti-Jo1 negative, those with better parameters in terms of respiratory function and significantly greater 6MWT presented moderate positive correlations between the relative abundance levels of *Veillonella* and *Streptococcus*. It should be noted that certain species of *Streptococcus* produce hydrogen peroxide, which can be used by *Veillonella*, thus generating a reducing microenvironment that improves the conditions for the establishment of other bacteria that are unable to tolerate an environment with reactive oxygen species (Zhou et al., 2017). Furthermore, a less inflammatory microenvironment could be generated due to the abundance of *Veillonella* in anti-Jo1-negative patients. This could explain the finding of the strong correlation between the abundance of *Streptococcu*s and DLCO (p<0.001, rho=-0.95). We did not find a strong correlation and statistically significant differences between FVC%, FEV1%, DLCO%, or 6MWT and *Veillonella*, *Sphingomonas*, *Prevotella*, and *Sediminebacterium*.

When we evaluated *Veillonella parvula, atypica*, and *dispar* species, there were no statistically significant differences between the study groups; however, *V. dispar* was less abundant in the JoP patients group than in the NJo group. *Veillonella* has been associated with different diseases, such as asthma (Espuela-Ortiz et al., 2019), COPD, and IPF exacerbations (Molyneaux et al., 2017).

Several reports conclude that the presence of *Veillonell*a is good for the health of humans. In patients with non-small cell lung cancer, *V. dispar* is predominated in groups with high levels of programmed cell death-ligand 1 (PD-L1), which are responders to immunotherapy, proving that the composition of the lung microbiome may influence disease development as well as treatment efficacy (Jang et al., 2021).

In evaluating the gut microbiome of elite athletes, the presence of *V. atypica* improves runtime via its metabolic conversion of exercise-induced lactate into propionate, thereby identifying a natural enzymatic process that enhances athletic performance (Scheiman et al., 2019). In diseases related to chronic hypoxemia, there is a high metabolism of lactate (Iscra et al., 2002), which *Veillonella* sp. could metabolize and shows a similar effect as that at the intestinal level; these findings could explain why patients included in this study who had a higher abundance of *Veillonella* exhibited more time walking in the 6MWT.

The knowledge of the bacterial communities in patients with ASSD, as well as the interaction mechanisms related to the immune response and remodeling of the pulmonary epithelium, will allow the identification of possible bacterial genera as biomarkers of ASSD, with the purpose of a better the understanding, management, and treatment of ASSD-ILD. In patients with ASSD, greater caspase-1, and higher LDH activity were observed in BAL, suggesting cell death due to pyroptosis and activation of the inflammasome pathway (Ramos-Martinez et al., 2022). Serum levels of 18 cytokines from baseline and after six months of treatment were quantified in patients with anti-tRNA associated ILD (anti-tRNA-ILD) and estimated the association between these and ILD improvement and progression; patients were classified as with or without ILD progression at six months. Only three patients had ILD progression (progressors patients, PP) and showed statistically higher levels in IL-4, IL-10, IL-17A, IL-22, GM-CSF, IL-1β, IL-6, IL-12, IL-18, and TNF-α, compared to patients without disease progression (no progressors patients, NPP). IL-17A, IL-1β, and IL-6 (T-helper-lymphocyte (Th)17 inflammatory cytokine profile) were elevated and had a high discriminatory capacity in distinguishing ILD PP of those NPP at follow-up (Ramos-Martinez et al., 2020).

Our findings show that the composition of the lung microbiome is different according to the profile of autoantibodies present in patients with ASSD-ILD, and in turn, there are correlations between bacterial genera and cells in the BAL that depend on anti-Jo1 autoantibodies. The methodology used to evaluate the lung microbiome only allows the exact identification up to bacterial genus. Our results provide evidence that patients with ASSD and positive anti-Jo1 autoantibodies have decreased *Veillonella* abundance, correlated with an increase in the count of lymphocytes, eosinophils, and other bacteria relevant to lung diseases.

The main limitation of this study was the lack of a control group. In the literature, there are reports about lung microbiome composition in healthy subjects. Firmicutes and Bacteroidota are the predominant phyla in healthy lung microbiota, with *Prevotella*, *Veillonella*, and *Streptococcus* being the most common genera (Mathieu et al., 2018; Yi et al., 2022). Pathological conditions lead to a loss of that diversity, with increased concentrations of some bacterial genera (Costa et al., 2018). We found a low abundance of *Veillonella* in patients with ASDD and anti-Jo1 autoantibodies; however, because of the heterogeneity of the lung microbiome between individuals, the characterization of the lung microbiome should be verified in a larger study.

## 5 Conclusion

The lung microbiome in ASSD patients with anti-Jo1 autoantibodies showed a low abundance of *Veillonella* compared to non-Jo1-positive ASSD patients. This genus had a strong and positive correlation with *Prevotella* abundance and levels of eosinophils and lymphocytes, and it showed a strong negative correlation with the percentage of macrophages. These correlations were not observed in non-Jo1-positive patients.

## Data availability statement

The datasets presented in this study can be found in online repositories. The names of the repository/repositories and accession number(s) can be found below: https://www.ncbi.nlm.nih.gov/, BioProject accession code PRJNA715451.

## Ethics statement

The Institutional Committee on Ethics and Research reviewed and approved the study (ethical protocol number B28-18). The studies were conducted in accordance with the local legislation and institutional requirements. The participants provided their written informed consent to participate in this study.

## Author contributions

TQ-P: Formal analysis, Resources, Writing – original draft. JL-L: Methodology, Software, Writing – original draft. RF-V: Funding acquisition, Supervision, Validation, Writing – review & editing. ÁV-S: Data curation, Validation, Writing – review & editing. EM-G: Resources, Validation, Writing – review & editing. MM: Supervision, Visualization, Writing – review & editing. BB-B: Formal analysis, Investigation, Writing – review & editing. JR-S: Validation, Visualization, Writing – review & editing. ER-M: Data curation, Writing – review & editing. IB-R: Data curation, Visualization, Writing – review & editing. GP-R: Formal analysis, Investigation, Project administration, Writing – original draft.

## References

[B1] Aggarwal R. Cassidy E. Fertig N. Koontz D. C. Lucas M. Ascherman D. P.. (2014). Patients with non-Jo-1 anti-tRNA-synthetase autoantibodies have worse survival than Jo-1 positive patients. Ann. Rheum Dis. 73 (1), 227–232. doi: 10.1136/annrheumdis-2012-201800 23422076 PMC4031026

[B2] Asghar M. T. Khurshid M. Nazir J. Shakoori A. R. (2021). Induction of apoptosis in human lung epithelial cell by sphingomonas sp. Shah, a recently identified cell culture contaminant. Crit. Rev. Eukaryot Gene Expr. 31 (2), 55–62. doi: 10.1615/CritRevEukaryotGeneExpr.2021037677 34347979

[B3] (2021) BLAST: Basic Local Alignment Search Tool. Available at: https://blast.ncbi.nlm.nih.gov/Blast.cgi.

[B4] Callahan B. J. McMurdie P. J. Rosen M. J. Han A. W. Johnson A. J. Holmes S. P. (2016a). DADA2: High-resolution sample inference from Illumina amplicon data. Nat. Methods 13 (7), 581–583. doi: 10.1038/nmeth.3869 27214047 PMC4927377

[B5] Callahan B. J. Sankaran K. Fukuyama J. A. McMurdie P. J. Holmes S. P. (2016b). Bioconductor Workflow for Microbiome Data Analysis: from raw reads to community analyses. F1000Res 5, 1492. doi: 10.12688/f1000research.8986.2 27508062 PMC4955027

[B6] Castro Nallar E. (2020). Introducción al análisis de diversidad | Diseño experimental y análisis de datos (Santiago, Chile: Castro Lab). Available at: http://www.castrolab.org/teaching/data_analysis/intro-analisis-diversidad.html.

[B7] Cavagna L. Trallero-Araguás E. Meloni F. Cavazzana I. Rojas-Serrano J. Feist E.. (2019). Influence of antisynthetase antibodies specificities on antisynthetase syndrome clinical spectrum time course. J. Clin. Med. 8 (11), 2013. doi: 10.3390/jcm8112013 31752231 PMC6912490

[B8] Costa A. N. Costa F. M. D. Campos S. V. Salles R. K. Athanazio R. A. (2018). The pulmonary microbiome: challenges of a new paradigm. J. Bras. Pneumol. 44 (5), 424–432. doi: 10.1590/s1806-37562017000000209 30066739 PMC6467588

[B9] Dickson R. P. Erb-Downward J. R. Martinez F. J. Huffnagle G. B. (2016). The microbiome and the respiratory tract. Annu. Rev. Physiol. 78, 481–504. doi: 10.1146/annurev-physiol-021115-105238 26527186 PMC4751994

[B10] Espuela-Ortiz A. Lorenzo-Diaz F. Baez-Ortega A. Eng C. Hernandez-Pacheco N. Oh S. S.. (2019). Bacterial salivary microbiome associates with asthma among african american children and young adults. Pediatr. Pulmonol. 54 (12), 1948–1956. doi: 10.1002/ppul.24504 31496123 PMC6851413

[B11] Gasparotto M. Gatto M. Saccon F. Ghirardello A. Iaccarino L. Doria A. (2019). Pulmonary involvement in antisynthetase syndrome. Curr. Opin. Rheumatol. 31 (6), 603–610. doi: 10.1097/BOR.0000000000000663 31503025

[B12] González-Pérez M. I. Mejía-Hurtado J. G. Pérez-Román D. I. Buendía-Roldán I. Mejía M. Falfán-Valencia R.. (2020). Evolution of pulmonary function in a cohort of patients with interstitial lung disease and positive for antisynthetase antibodies. J. Rheumatol. 47 (3), 415–423. doi: 10.3899/jrheum.181141 31203227

[B13] Hamaguchi Y. Fujimoto M. Matsushita T. Kaji K. Komura K. Hasegawa M.. (2013). Common and distinct clinical features in adult patients with anti-aminoacyl-tRNA synthetase antibodies: heterogeneity within the syndrome. PloS One 8 (4), e60442. doi: 10.1371/journal.pone.0060442 23573256 PMC3616126

[B14] Han M. K. Zhou Y. Murray S. Tayob N. Noth I. Lama V. N.. (2014). Lung microbiome and disease progression in idiopathic pulmonary fibrosis: an analysis of the COMET study. Lancet Respir. Med. 2 (7), 548–556. doi: 10.1016/S2213-2600(14)70069-4 24767767 PMC4142525

[B15] Huffnagle G. B. Dickson R. P. Lukacs N. W. (2017). The respiratory tract microbiome and lung inflammation: a two-way street. Mucosal Immunol. 10 (2), 299–306. doi: 10.1038/mi.2016.108 27966551 PMC5765541

[B16] Iscra F. Gullo A. Biolo G. (2002). Bench-to-bedside review: lactate and the lung. Crit. Care 6 (4), 327–329. doi: 10.1186/cc1519 12225608 PMC137459

[B17] Jang H. J. Choi J. Y. Kim K. Yong S. H. Kim Y. W. Kim S. Y.. (2021). Relationship of the lung microbiome with PD-L1 expression and immunotherapy response in lung cancer. Respir. Res. 22 (1), 322. doi: 10.1186/s12931-021-01919-1 34963470 PMC8715618

[B18] Kamiya H. Panlaqui O. M. Izumi S. Sozu T. (2018). Systematic review and meta-analysis of prognostic factors for idiopathic inflammatory myopathy-associated interstitial lung disease. BMJ Open 8 (12), e023998. doi: 10.1136/bmjopen-2018-023998 PMC6303632 30559160

[B19] Lahti L. Sudarshan S.. (2017) Introduction to the microbiome R package [Internet]. Bioconductor. Available at: https://microbiome.github.io/tutorials/.

[B20] Lang D. Akbari K. Horner A. Hepp M. Kaiser B. Pieringer H.. (2021). Computed tomography findings as determinants of local and systemic inflammation biomarkers in interstitial lung diseases: A retrospective registry-based descriptive study. Lung 199 (2), 155–164. doi: 10.1007/s00408-021-00434-w 33770227 PMC8053160

[B21] Lee H. K. Kim D. S. Yoo B. Seo J. B. Rho J. Y. Colby T. V.. (2005). Histopathologic pattern and clinical features of rheumatoid arthritis-associated interstitial lung disease. Chest 127 (6), 2019–2027. doi: 10.1378/chest.127.6.2019 15947315

[B22] Lega J. C. Cottin V. Fabien N. Thivolet-Béjui F. Cordier J. F. (2010). Interstitial lung disease associated with anti-PM/Scl or anti-aminoacyl-tRNA synthetase autoantibodies: a similar condition? J. Rheumatol 37 (5), 1000–1009. doi: 10.3899/jrheum.090652 20231208

[B23] Lin Pedersen T. (2019) Package “ggplot2” Title Create Elegant Data Visualisations Using the Grammar of Graphics. Available at: https://cran.r-project.org/web/packages/ggplot2/ggplot2.pdf.

[B24] Liu Y. Liu X. Xie M. Chen Z. He J. Wang Z.. (2020). Clinical characteristics of patients with anti-EJ antisynthetase syndrome associated interstitial lung disease and literature review. Respir. Med. 165, 105920. doi: 10.1016/j.rmed.2020.105920 32174452

[B25] Lou Y. Wei Q. Fan B. Zhang L. Wang X. Chen Z.. (2022). The composition of the lung microbiome differs between patients with dermatomyositis and rheumatoid arthritis associated with interstitial lung disease. FEBS Open Bio 12 (1), 258–269. doi: 10.1002/2211-5463.13334 PMC8727938 34800087

[B26] Love M. I. Huber W. Anders S. (2014). Moderated estimation of fold change and dispersion for RNA-seq data with DESeq2. Genome Biol. 15 (12), 550. doi: 10.1186/s13059-014-0550-8 25516281 PMC4302049

[B27] Marie I. Hatron P. Y. Cherin P. Hachulla E. Diot E. Vittecoq O.. (2013a). Functional outcome and prognostic factors in anti-Jo1 patients with antisynthetase syndrome. Arthritis Res. Ther. 15 (5), R149. doi: 10.1186/ar4332 24286268 PMC3978997

[B28] Marie I. Josse S. Hatron P. Y. Dominique S. Hachulla E. Janvresse A.. (2013b). Interstitial lung disease in anti-Jo-1 patients with antisynthetase syndrome. Arthritis Care Res. (Hoboken). 65 (5), 800–808. doi: 10.1002/acr.21895 23203765

[B29] Mathieu E. Escribano-Vazquez U. Descamps D. Cherbuy C. Langella P. Riffault S.. (2018). Paradigms of lung microbiota functions in health and disease, particularly, in asthma. Front. Physiol. 9, 1168. doi: 10.3389/fphys.2018.01168 30246806 PMC6110890

[B30] McMurdie P. J. Holmes S. (2013). phyloseq: an R package for reproducible interactive analysis and graphics of microbiome census data. PloS One 8 (4), e61217. doi: 10.1371/journal.pone.0061217 23630581 PMC3632530

[B31] Mirković B. Murray M. A. Lavelle G. M. Molloy K. Azim A. A. Gunaratnam C.. (2015). The role of short-chain fatty acids, produced by anaerobic bacteria, in the cystic fibrosis airway. Am. J. Respir. Crit. Care Med. 192 (11), 1314–1324. doi: 10.1164/rccm.201505-0943OC 26266556 PMC4731701

[B32] Molyneaux P. L. Cox M. J. Wells A. U. Kim H. C. Ji W. Cookson W. O.. (2017). Changes in the respiratory microbiome during acute exacerbations of idiopathic pulmonary fibrosis. Respir. Res. 18 (1), 29. doi: 10.1186/s12931-017-0511-3 28143484 PMC5286769

[B33] Neish A. S. (2014). Mucosal immunity and the microbiome. Ann. Am. Thorac. Soc 11 Suppl 1 (Suppl 1), S28–S32. doi: 10.1513/AnnalsATS.201306-161MG 24437401 PMC3972979

[B34] O’Dwyer D. N. Ashley S. L. Gurczynski S. J. Xia M. Wilke C. Falkowski N. R.. (2019). Lung microbiota contribute to pulmonary inflammation and disease progression in pulmonary fibrosis. Am. J. Respir. Crit. Care Med. 199 (9), 1127–1138. doi: 10.1164/rccm.201809-1650OC 30789747 PMC6515865

[B35] Paudel K. R. Dharwal V. Patel V. K. Galvao I. Wadhwa R. Malyla V.. (2020). Role of lung microbiome in innate immune response associated with chronic lung diseases. Front. Med. (Lausanne). 7, 554. doi: 10.3389/fmed.2020.00554 33043031 PMC7530186

[B36] Pinal-Fernandez I. Casal-Dominguez M. Huapaya J. A. Albayda J. Paik J. J. Johnson C.. (2017). A longitudinal cohort study of the anti-synthetase syndrome: increased severity of interstitial lung disease in black patients and patients with anti-PL7 and anti-PL12 autoantibodies. Rheumatol. (Oxford). 56 (6), 999–1007. doi: 10.1093/rheumatology/kex021 PMC5850781 28339994

[B37] Ponce M. C. Sankari A. Sharma S. (2022). “Pulmonary Function Tests,” in StatPearls (Treasure Island (FL: StatPearls Publishing). 29493964

[B38] Ponce-Gallegos M. A. Ramos-Martínez E. García-Carmona A. Mejía M. Nava-Quiroz K. J. Pérez-Rubio G.. (2020). Genetic susceptibility to antisynthetase syndrome associated with single-nucleotide variants in the IL1B gene that lead variation in IL-1β Serum levels. Front. Med. (Lausanne). 7, 547186. doi: 10.3389/fmed.2020.547186 33330522 PMC7732678

[B39] Qiu J. Zhou H. Jing Y. Dong C. (2019). Association between blood microbiome and type 2 diabetes mellitus: A nested case-control study. J. Clin. Lab. Anal. 33 (4), e22842. doi: 10.1002/jcla.22842 30714640 PMC6528574

[B40] Quast C. Pruesse E. Yilmaz P. Gerken J. Schweer T. Yarza P.. (2013). The SILVA ribosomal RNA gene database project: improved data processing and web-based tools. Nucleic Acids Res. 41 (Database issue), D590–D596. doi: 10.1093/nar/gks1219 23193283 PMC3531112

[B41] Ramos-Martinez E. Falfán-Valencia R. Pérez-Rubio G. Mejia M. Buendía-Roldán I. González-Pérez M. I.. (2020). Anti-aminoacyl transfer-RNA-synthetases (Anti-tRNA) autoantibodies associated with interstitial lung disease: pulmonary disease progression has a persistent elevation of the th17 cytokine profile. J. Clin. Med. 9 (5), 1356. doi: 10.3390/jcm9051356 32384594 PMC7290669

[B42] Ramos-Martinez E. Vega-Sánchez A. E. Pérez-Rubio G. Mejia M. Buendía-Roldán I. González-Pérez M. I.. (2022). Enhanced activity of NLRP3 inflammasome in the lung of patients with anti-synthetase syndrome. Cells 12 (1), 60. doi: 10.3390/cells12010060 36611853 PMC9818379

[B43] Ren L. Zhang R. Rao J. Xiao Y. Zhang Z. Yang B.. (2018). Transcriptionally active lung microbiome and its association with bacterial biomass and host inflammatory status. mSystems 3 (5), e00199–e00118. doi: 10.1128/mSystems.00199-18 30417108 PMC6208642

[B44] Rojas-Serrano J. Herrera-Bringas D. Mejía M. Rivero H. Mateos-Toledo H. Figueroa J. E. (2015). Prognostic factors in a cohort of antisynthetase syndrome (ASS): serologic profile is associated with mortality in patients with interstitial lung disease (ILD). Clin. Rheumatol. 34 (9), 1563–1569. doi: 10.1007/s10067-015-3023-x 26219488

[B45] R Studio (2019) RStudio | Open source & professional software for data science teams - RStudio [Internet]. Available at: https://www.rstudio.com/.

[B46] Scheiman J. Luber J. M. Chavkin T. A. MacDonald T. Tung A. Pham L. D.. (2019). Meta-omics analysis of elite athletes identifies a performance-enhancing microbe that functions via lactate metabolism. Nat. Med. 25 (7), 1104–1109. doi: 10.1038/s41591-019-0485-4 31235964 PMC7368972

[B47] Scher J. U. Joshua V. Artacho A. Abdollahi-Roodsaz S. Öckinger J. Kullberg S.. (2016). The lung microbiota in early rheumatoid arthritis and autoimmunity. Microbiome 4 (1), 60. doi: 10.1186/s40168-016-0206-x 27855721 PMC5114783

[B48] Segal L. N. Clemente J. C. Tsay J. C. Koralov S. B. Keller B. C. Wu B. G.. (2016). Enrichment of the lung microbiome with oral taxa is associated with lung inflammation of a Th17 phenotype. Nat. Microbiol. 1, 16031. doi: 10.1038/nmicrobiol.2016.31 27572644 PMC5010013

[B49] Soehnlein O. Oehmcke S. Ma X. Rothfuchs A. G. Frithiof R. van Rooijen N.. (2008). Neutrophil degranulation mediates severe lung damage triggered by streptococcal M1 protein. Eur. Respir. J. 32 (2), 405–412. doi: 10.1183/09031936.00173207 18321926

[B50] Tunney M. M. Einarsson G. G. Wei L. Drain M. Klem E. R. Cardwell C.. (2013). Lung microbiota and bacterial abundance in patients with bronchiectasis when clinically stable and during exacerbation. Am. J. Respir. Crit. Care Med. 187 (10), 1118–1126. doi: 10.1164/rccm.201210-1937OC 23348972 PMC3734618

[B51] Vinolo M. A. Ferguson G. J. Kulkarni S. Damoulakis G. Anderson K. Bohlooly-Y M.. (2011). SCFAs induce mouse neutrophil chemotaxis through the GPR43 receptor. PloS One 6 (6), e21205. doi: 10.1371/journal.pone.0021205 21698257 PMC3115979

[B52] Vinolo M. A. Hatanaka E. Lambertucci R. H. Newsholme P. Curi R. (2009). Effects of short chain fatty acids on effector mechanisms of neutrophils. Cell Biochem. Funct. 27 (1), 48–55. doi: 10.1002/cbf.1533 19107872

[B53] Wei T. Simko V. (2021) R package “corrplot”: Visualization of a Correlation Matrix. Available at: https://github.com/taiyun/corrplot.

[B54] Wickham H. (2016). ggplot2: Elegant Graphics for Data Analysis (New York: Springer-Verlag). Available at: https://ggplot2.tidyverse.org.

[B55] Witt L. J. Curran J. J. Strek M. E. (2016). The diagnosis and treatment of antisynthetase syndrome. Clin. Pulm Med. 23 (5), 218–226. doi: 10.1097/CPM.0000000000000171 27594777 PMC5006392

[B56] Yagi K. Huffnagle G. B. Lukacs N. W. Asai N. (2021). The lung microbiome during health and disease. Int. J. Mol. Sci. 22 (19), 10872. doi: 10.3390/ijms221910872 34639212 PMC8509400

[B57] Yi X. Gao J. Wang Z. (2022). The human lung microbiome—A hidden link between microbes and human health and diseases. iMeta 1, e33. doi: 10.1002/imt2.33 PMC10989958 38868714

[B58] Yu G. Gail M. H. Consonni D. Carugno M. Humphrys M. Pesatori A. C.. (2016). Characterizing human lung tissue microbiota and its relationship to epidemiological and clinical features. Genome Biol. 17 (1), 163. doi: 10.1186/s13059-016-1021-1 27468850 PMC4964003

[B59] Zamora A. C. Hoskote S. S. Abascal-Bolado B. White D. Cox C. W. Ryu J. H.. (2016). Clinical features and outcomes of interstitial lung disease in anti-Jo-1 positive antisynthetase syndrome. Respir. Med. 118, 39–45. doi: 10.1016/j.rmed.2016.07.009 27578469

[B60] Zhou P. Li X. Huang I. H. Qi F. (2017). *Veillonella* Catalase Protects the Growth of Fusobacterium nucleatum in Microaerophilic and *Streptococcus gordonii-*Resident Environments. Appl. Environ. Microbiol. 83 (19), e01079–e01017. doi: 10.1128/AEM.01079-17 28778894 PMC5601340

