# Supplementary material for: Lung microbiome alterations in patients with anti-Jo1 antisynthetase syndrome and interstitial lung disease
Source: Front Cell Infect Microbiol. 2023 Dec 5;13:1321315. doi: 10.3389/fcimb.2023.1321315 (PMC10728596; doi:10.3389/fcimb.2023.1321315)
Supplement: Supplementary file 1 [file DataSheet_1.docx]

Supplementary Material

**Lung microbiome alterations in patients with anti-Jo1 antisynthetase syndrome and interstitial lung disease**

**Teresa Quintero-Puerta ^1^, Juan Alberto Lira-Lucio ^1^, Ramcés Falfán-Valencia ^1^, Ángel E Vega-Sánchez ^2^, Eduardo Márquez-García ^3^, Mayra Mejía ^2^, Brandon Bautista-Becerril ^1^, Jorge Rojas-Serrano ^2^, Espiridión Ramos-Martínez ^4^, Ivette Buendía-Roldán ^5^ and Gloria Pérez-Rubio ^1*^**

^1^HLA Laboratory, Instituto Nacional de Enfermedades Respiratorias Ismael Cosío Villegas, Mexico City 14080, Mexico

^2^Interstitial Lung Disease and Rheumatology Unit, Instituto Nacional de Enfermedades Respiratorias Ismael Cosío Villegas, Mexico City 14080, Mexico.

^3^Subdirección de Investigación Biomédica, Instituto Nacional de Enfermedades Respiratorias Ismael Cosío Villegas, Mexico City, Mexico.

^4^Experimental Medicine Research Unit, Facultad de Medicina, Universidad Nacional Autónoma de México 06720 México City Mexico.

^5^Laboratory of Translational Research in Aging and Pulmonary, Instituto Nacional de Enfermedades Respiratorias Ismael Cosío Villegas, Mexico City, Mexico.

*** Correspondence:**Gloria Pérez-Rubio
[gperezrubio@iner.gob.mx](mailto:gperezrubio@iner.gob.mx)

**Table S1. Primers and probe sequences of *Veillonella***

| ***Veillonella*** | **Primers and probe sequences** |
| --- | --- |
| *parvula* (X84005.1) (33) | Forward: 5´-TGCTAATACCGCATACGATCTAACC-3´  Reverse: 5´- GCTTATAAATAGAGGCCACCTTTCA-3´  Probe: **VIC**- CTATCCTCGATGCCGA |
| *dispar* (34,35) | Forward 5´- CTACAATGGGAGTTAATAGACGGAAG-3´  Reverse 5´- CAGCCTACGATCCGAACTGAG-3´  Probe **FAM**-AGCAAACCCGAGAAACACT |
| *atypica (NR_041880.1)* (custom design) | Forward: 5´- ATACGTAGGTGGCAAGCGTT-3´  Reverse: 5´- ACGCATTTCACCGCTACACT-3´  Maxima **SYBR Green**/ROX |

**Table S2. Clinical manifestations in the study sample.**

| **Variable** | **All patients, n= 23 (%)** | **JoP,**  **n = 6 (%)** | **NJo,**  **n = 17 (%)** | **p-value** |
| --- | --- | --- | --- | --- |
| Signs of mechanic’s hand | 10 (43.5) | 3 (50.0) | 7 (41.7) | 1.00 |
| Arthritis | 1 (4.3) | 0 | 1 (5.8) | *NA* |
| Arthralgia | 7 (30.5) | 1 (16.7) | 6 (35.3) | 0.62 |
| Myalgia | 4 (17.4) | 1 (16.7) | 3 (17.6) | 1.00 |
| Dyspnea | 10 (43.5) | 2 (33.3) | 8 (47.0) | 0.66 |
| Cough | 10 (43.5) | 2 (33.3) | 8 (47.0) | 0.66 |
| Fever | 6 (26.0) | 0 | 6 (35.3) | *NA* |
| Weight loss | 4 (17.4) | 0 | 4 (23.5) | *NA* |
| Muscular weakness | 2 (8.7) | 1 (16.7) | 1 (5.8) | 0.46 |
| Xerostomia | 5 (21.7) | 0 | 5 (29.4) | *NA* |

JoP, anti-Jo1-positive patients; NJo, non-Jo1-positive patients (NJo). **The values in the parentheses are percentages.**

The p-value obtained using Fisher's exact test was used to compare the groups.

**Table S3. Antibody profile in the study sample.**

| Antibodies | JoP (n=6) | NJo (n=17) |
| --- | --- | --- |
| Ro52, % | 16.6 | 35.3 |
| PL7, % | 16.6 | 35.3 |
| PL12, % | 0 | 35.3 |
| OJ, % | 33.3 | 29.4 |
| EJ, % | 0 | 11.7 |
| MI-2a, % | 0 | 17.6 |
| MI-2b, % | 33.3 | 11.7 |
| SRP, % | 0 | 29.4 |
| NXP-2, % | 0 | 23.5 |
| TIF-1γ, % | 16.6 | 5.9 |
| SAE1, % | 0 | 11.7 |
| MDA-5, % | 0 | 5.8 |
| Th/To, % | 33.3 | 23.5 |
| Ku, % | 0 | 17.6 |
| PM/Scl-70, % | 0 | 5.8 |
| PM/Scl-100, % | 0 | 11.7 |

JoP, anti-Jo1-positive patients; NJo, non-Jo1-positive patients (NJo). The values are presented as percentages.

The p-value obtained using Fisher's exact test was used to compare the groups.


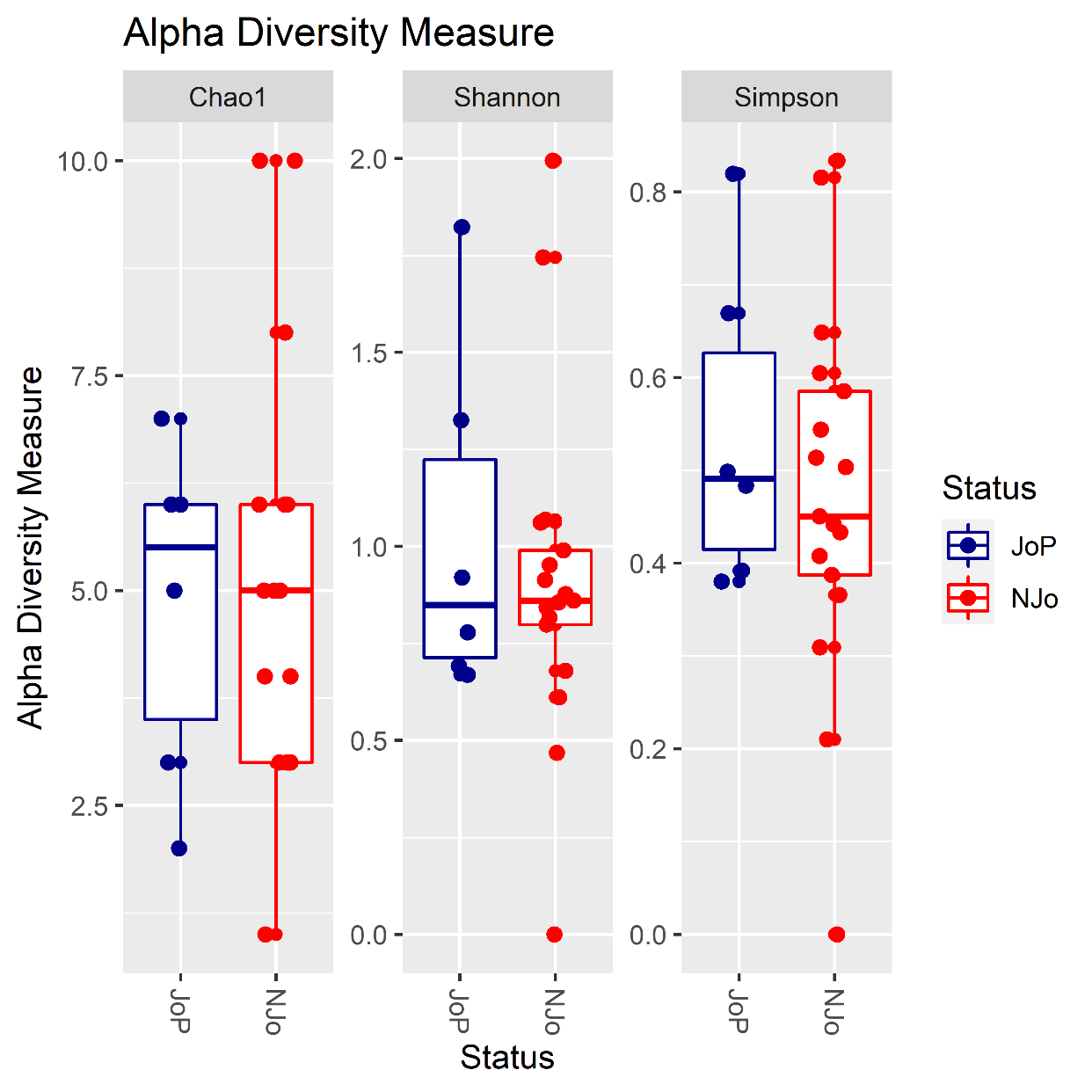


Figure S1. Alpha diversity.


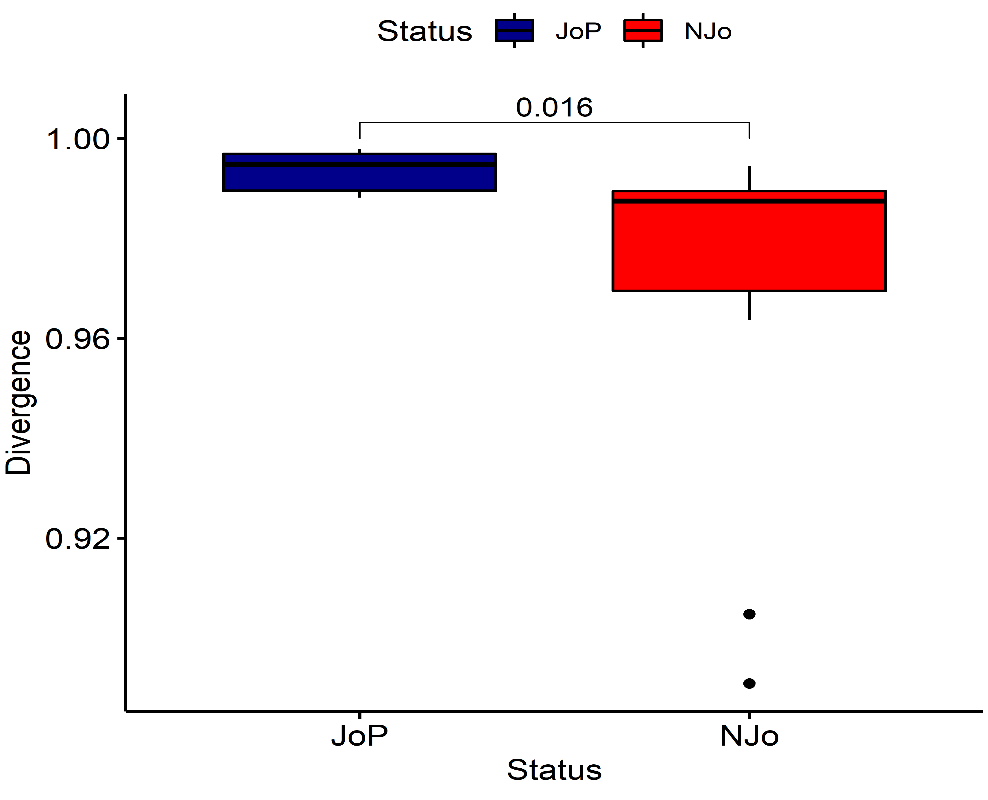


Figure S2a. Beta diversity: divergence between the groups included in the study.


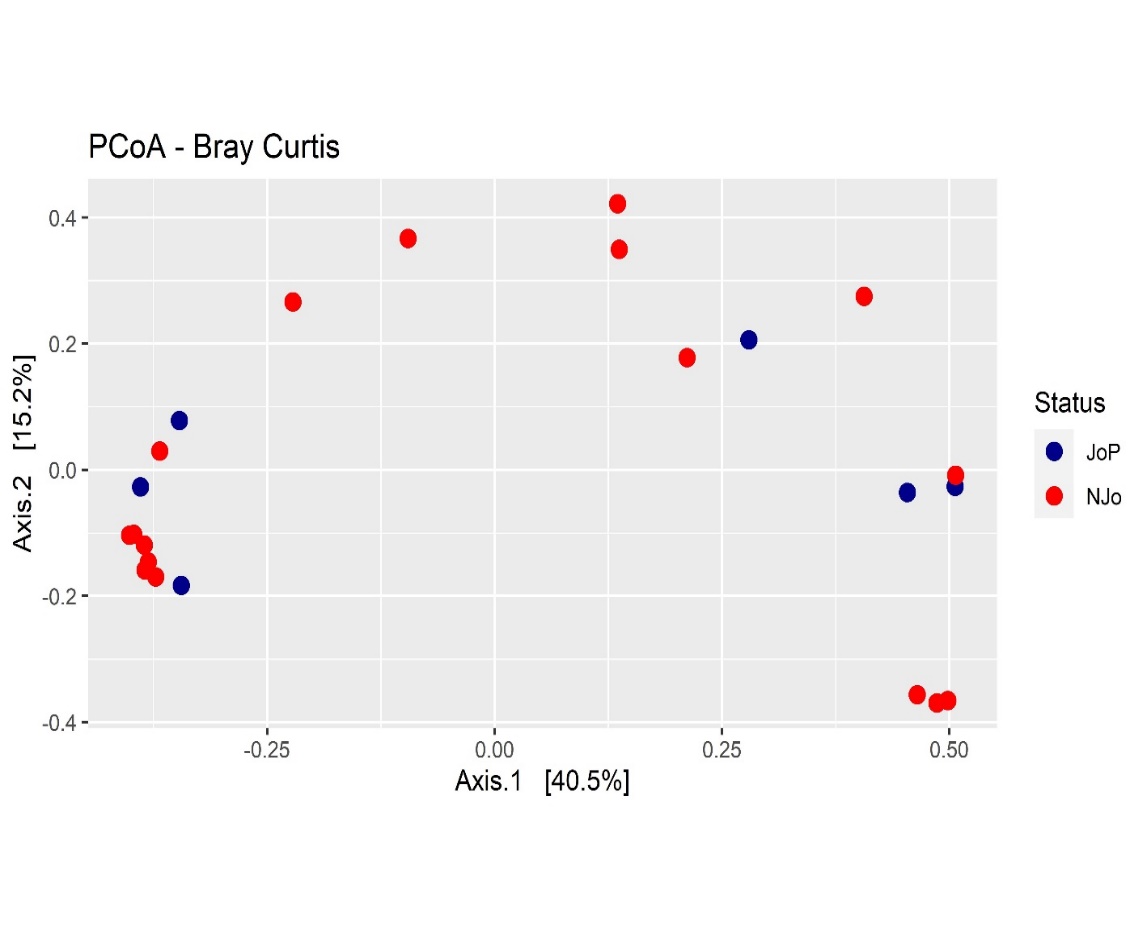


Figure S2b. PCoA between JoP and NJo groups.
